# Arguments for the choice of surgical treatments in patients with lumbar spinal stenosis – a systematic appraisal of randomized controlled trials

**DOI:** 10.1186/s12891-015-0548-8

**Published:** 2015-04-22

**Authors:** Jakob M Burgstaller, François Porchet, Johann Steurer, Maria M Wertli

**Affiliations:** Horten Centre for Patient Oriented Research and Knowledge Transfer, Department of Internal Medicine, University of Zurich, Pestalozzistrasse 24, 8032 Zurich, Switzerland; Department of Neurosurgery, Spine Center, Schulthess Clinic, Zürich, Switzerland

**Keywords:** Lumbar spinal stenosis, Surgery, Decompression, Decompression and fusion, Fecompression and fusion with instrumentation, Systematic review, Randomized controlled trial, Instability, Reoperation, Low back pain

## Abstract

**Background:**

Lumbar spinal stenosis is the most common reason for spinal surgery in elderly patients. However, the surgical management of spinal stenosis is controversial. The aim of this review was to list aspects a surgeon considers when choosing a specific type of treatment.

**Methods:**

Appraisal of arguments reported in randomized controlled trials (RCTs) included in systematic reviews published or indexed in the Cochrane library studying surgical treatments in patients with spinal stenosis.

**Results:**

Eight out of nine RCTs listed arguments for the choice of their treatments under investigation. The argument for decompression alone was the high success rate, the argument against was a potential increase in vertebral instability. The argument for decompression and fusion without instrumentation was that it is a well-established technique with a high fusion success rate, the argument against it was that the indication for fusion in spinal stenosis has remained unclear. The argument for decompression and fusion with instrumentation was an increased fusion rate compared to decompression and fusion without instrumentation, the argument against this was that the invasive procedure is associated with more complications.

**Conclusions:**

The main argument identified in this appraisal for and against decompression alone in patient with lumbar spinal stenosis was whether or not instability should be treated with (instrumented) fusion procedures. However, there is disagreement on how instability should be defined. In a first step it is important that researchers and clinicians agree on definitions for important key concepts such as instability and reoperations.

**Electronic supplementary material:**

The online version of this article (doi:10.1186/s12891-015-0548-8) contains supplementary material, which is available to authorized users.

## Background

The clinical entity lumbar spinal stenosis is the most common reason for spinal surgery in patients 65 years of age and older in the United States [1]. The clinical definition includes “buttock or lower extremity pain, which may occur with or without low back pain, associated with diminished space available for the neural and vascular elements in the lumbar spine” [2]. Herniated intervertebral discs and space-narrowing lesions caused by neoplasm or inflammation are in the strictest sense also causes of stenosis, but they usually are regarded as separate entities [3]. Neurogenic claudication (pain in the legs during walking with or without low back pain) is the main complaint of patients, caused by increased compression of intracanalar nervous structures. It has been shown that symptoms often poorly correlate with imaging studies [4].

The management of spinal stenosis is still controversial. For mild symptoms conservative treatment seems to be the natural choice although controlled clinical studies comparing conservative and surgical treatment are rare and little is known about the short- and long-term course of this approach [3]. When symptoms are severe and conservative treatment has failed, surgery is suggested. The type of surgery that should be performed remains also controversial [5]. Decompression seems to be the logical procedure that has the potential to give the patient immediate relief [3]. However, instability of the spine is a potential consequence that needs to be considered. The additional value of decompression and arthrodesis compared to decompression is debated [5]. It is still unclear which aspects and arguments influence the decision to choose the type of surgical treatment for a patient with spinal stenosis.

Therefore, the aim of this systematic appraisal of arguments for or against a type of surgical treatment was to list aspects a surgeon considers when choosing a specific type of treatment for a patient.

## Methods

### Study design

Appraisal of arguments reported in randomized controlled trials (RCTs) included in systematic reviews published or indexed in the Cochrane library studying surgical treatments in patients with spinal stenosis. The Cochrane Collaboration Guideline has published guidelines for the standardized assessment of study quality in randomized controlled trials. Therefore, systematic reviews published or indexed in the Cochrane library meet high quality standards [6]. While this study is not a systematic review our reporting will be based on the recommendations of the PRISMA statement.

### Literature search

We searched the Cochrane library for the term “spinal stenosis” in the title, abstract, or keywords. Of the returned reviews, only RCTs that investigated efficacy of surgical treatments for patients with lumbar spinal stenosis were eligible for further analysis. Non-randomized trials and observational studies were excluded. In published study protocol of systematic reviews which were potentially eligible, the authors were contacted for additional information.

### Eligibility criteria

Included were all RCTs that studied efficacy of surgical treatments for lumbar spinal stenosis, No limits for the study setting or language of the publication were applied. Excluded were RCTs that reported about disc replacement procedures, intradiscal electrotherapy, or RCTs with a control group which was treated with conservative methods.

### Study selection, data extraction and data synthesis

The bibliographic details of all retrieved articles were stored in an Endnote file. Two reviewers (JB and MW) independently screened all systematic reviews by title and abstract. All potentially eligible RCTs were included for the full text analysis. The full text was reviewed by both reviewers independently (JB and MW) in all RCTs that met the pre-defined eligibility criteria (n = 63). Alternative researchers with specific language proficiencies were used for non-English language references. Arguments in study reports were extracted by two reviewers independently (JB and MW). Disagreements were discussed and resolved by consensus or by third party arbitration (FP). Arguments were assigned to the following groups by one reviewer (JB): decompression, decompression and fusion without instrumentation (hereinafter abbreviated to ‘fusion’ or ‘fusion without instrumentation’), and decompression and fusion with instrumentation (hereinafter abbreviated to ‘fusion with instrumentation’ or ‘instrumented fusion’).

### Surgical procedures

Decompression is defined as “the relief of pressure on one or many pinched nerves (neural impingement) of the spinal column” [7]. Several different techniques are summarized under the term decompression: partial or total laminectomy, hemilaminectomy, laminotomy, and medial facetectomy.

Fusion, also known as spondylodesis, is defined as “a surgical technique used to join two or more vertebrae”. Bone graft, either from the patient (autograft), a donor (allograft), or bone substitute, is used in conjunction with the body's natural bone growth (osteoblastic) processes to fuse the vertebrae.

Fusion with instrumentation utilizes stainless steel, titanium (−alloy), or non-metallic devices to stabilize the spine.

Table 1 presents a more detailed overview over all surgical procedures.

**Table 1:** **Overview of the six systematic reviews and author’s conclusions**

**Author**	**Year**	**Objective**	**Author’s conclusion**
Chou D [29]	2011	To compare the effectiveness and morbidity of interspinous-device placement versus surgical decompression for the treatment of lumbar spinal stenosis.	The indirect treatment effect for disability and pain favors the interspinous device compared to decompression. The low evidence suggests that any further research is very likely to have an important impact on the confidence in the estimate of effect and is likely to change the estimate. No significant treatment effect differences were observed for postoperative walking distance improvement or complication rates; however, findings should be considered with caution because of indirect comparisons and short follow-up periods.
Gibson [30]	2005	The objective of this review was to assess current scientific evidence on the effectiveness of surgical interventions for degenerative lumbar spondylosis.	Limited evidence is now available to support some aspects of surgical practice. Surgeons should be encouraged to perform further RCTs in this field.
Jarrett [31]	2012	The aim of this review was to systematically examine the effectiveness of land based exercise compared with decompressive surgery in the management of patients with LSS.	This systematic review of the recent literature demonstrates that decompressive surgery is more effective than land based exercise in the management of LSS. However, given the condition’s slowly progressive nature and the potential for known surgical complications, it is recommended that a trial of conservative management with land based exercise be considered prior to consideration of surgical intervention.
Kovacs [32]	2011	To compare the effectiveness of surgery versus conservative treatment on pain, disability, and loss of quality of life caused by symptomatic lumbar spinal stenosis (LSS).	In patients with symptomatic LSS, the implantation of a specific type of device or decompressive surgery, with or without fusion, is more effective than continued conservative treatment when the latter has failed for 3 to 6 months.
May [8]	2013	To explore the effectiveness of surgery vs conservative treatment, and conservative interventions for spinal stenosis.	At present, there is no evidence that favours the effect of any conservative management for spinal stenosis.
Moojen [33]	2011	The main objective of this review was to perform a meta-analysis of all systematic reviews, randomized clinical trials and prospective cohort series to quantify the effectiveness of interspinous process distractions (IPDs) and to evaluate the potential side effects.	As the evidence is relatively low and the costs are high, more thorough (cost-) effectiveness studies should be performed before worldwide implementation is introduced.

#### Ethics statement

This study does not involve human subjects. No ethical approval was required. No protocol was published or registered. All methods were determined a priori.

## Results

### Study selection

**Figure 1:**
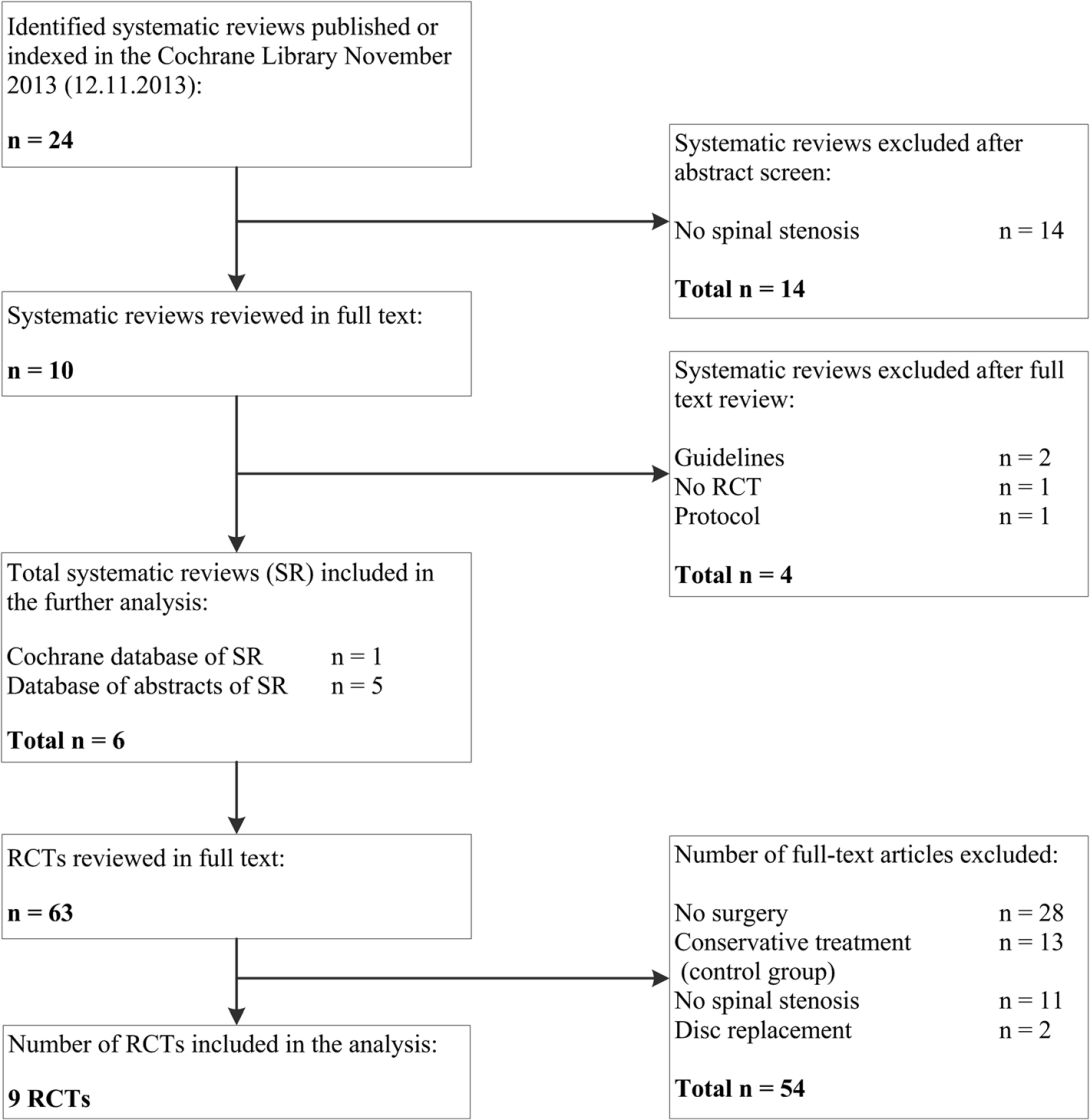
Study flow.

Figure 1 summarizes the search and inclusion process. Out of 24 systematic reviews, 6 systematic reviews were eligible for the current appraisal. Of the 6 systematic reviews 63 RCTs were reviewed in full text, resulting in exclusion of 54 RCTs. In total, the appraisal included 9 RCTs. Reasons for exclusion of 54 publications are given in Figure 1.

### Study characteristics

The most recent systematic review identified was published in 2013 [8]. Six systematic reviews addressed the efficacy of surgical treatments for lumbar spinal stenosis (Table 2). The characteristics of the RCTs included in the current appraisal and the surgical procedures under investigation as well as the definitions of instability are summarized in Additional file 1. Two RCTs compared posterolateral fusion with posterolateral instrumented fusion [9,10]. One RCT compared fusion and decompression [11]. One RCT compared instrumented fusion and decompression [12]. Two RCTs compared two different instrumented fusion procedures [13,14]. Two RCTs compared two decompression procedures [15,16]. One RCT compared fusion, instrumented fusion, and decompression [17]. Furthermore, only two RCTs [12,16] defined the term “instability”.

**Table 2:** **Overview of the different surgical procedures**

**Decompression**						
Laminectomy, partial or total	laminotomy	uni- or bilateral hemilaminotomy	medial facetectomy	foraminotomy	fenestration with undercutting decompression	
**Fusion promotors**						
Autologous bone transplantation	allogeneic bone transplantation	bone graft substitute(demineralized bone, ceramic extender, or bone morphogenetic protein)				
**Fusion technics**						
Anterior/posterior fusion	posterolateral fusion	circumferential fusion (360°, combined anterior interbody fusion and posterior fusion)	ALIF (anterior lumbar interbody fusion)	PLIF (posterior lumbar interbody fusion)	TLIF (transforaminal lumbar interbody fusion)	XLIF (extreme lateral interbody fusion)
**Fusion devices**						
Pedicle screws and rods	hooks and rods	pedicle screws and plates	anterior plates	interbody cages for:ALIF, PLIF, TLIF, XLIF		

The most recent RCT was conducted in 2005 (range of year of publications 1991–2005).

### Appraisal of arguments for or against a surgical technique

Of the 9 RCTs one provided no arguments for the choice of their treatments under investigation [17]. Of 8 RCTs arguments for or against a surgical technique were extracted. The arguments are summarized in Table 3 and grouped into arguments for or against decompression alone, fusion, and fusion with instrumentation.

**Table 3:** **Appraisal of arguments for and against a surgical technique**

**Decompression alone for spinal stenosis**			
**FOR**		**AGAINST**	
**Argument**	**Reference**	**Argument**	**Reference**
Bilateral and unilateral laminotomy for bilateral decompression: the success rates were as high as 90%.	[16]	increase or cause vertebral instability/spondylolisthesis progression after decompression alone + continuous motion of the stenotic segments may produce osteophytes as well as compression of the nerve roots	[12,15,16],
Satisfactory results with decompressive laminectomy alone.	[11]		
Results of simple decompression for degenerative spondylolisthesis have been excellent.	[13]		
**Decompression and fusion without instrumentation for spinal stenosis**			
**FOR**		**AGAINST**	
**argument**	**Reference**	**Argument**	**Reference**
Significant improvement in clinical outcome	[9]	controversy regarding the role of simultaneous arthrodesis of the spine: undisturbed relative stability of the decompressed spine can be maintained with meticulous operative technique	[12]
Results of posterolateral fusion for degenerative spondylolisthesis have been excellent.	[13]	it has been suggested that degenerative changes, such as osteophytes, decreased disc height, and calcified ligaments, increase the stability of the spine, thereby decreasing the need for an arthrodesis	[12]
Posterolateral fusion with only bone graft noted high fusion rates	[10]	Indications for fusion in degenerative lumbar spondylolisthesis and spinal stenosis have remained unclear	[11]
Noninstrumented posterolateral fusion has always been well-established and is done frequently	[10,14]		
**Decompression and fusion with instrumentation for spinal stenosis**			
**FOR**		**AGAINST**	
**Argument**	**Reference**	**Argument**	**Reference**
Improve fusion rate + prevent spondylolisthesis progressionMay improve fusion rate and may decrease rehabilitation time and may improve patient outcome	[9,10,14],	360° (circumferential) fusion: requires two surgeries, is expensive, and utilizes a great deal of health care resources	[14]
360° (circumferential) fusion: high fusion rate and a high level of patient satisfaction“270°” fusion (ALIF plus transpedicular instrumentation without PLF): may be effective	[14]	Complications: device-related osteoporosis	[10]
Semirigid systems have been advocated as a means of obtaining spinal stability without sacrificing vertebral body bone density	[10]		
Pedicle screw fixation increases rigidity despite resection of the posterior elements	[10]		

In three RCTs authors argued in favor of decompression alone compared to fusion with or without instrumentation. Three RCTs provided arguments against decompression alone. While four RCTs argued for fusion without instrumentation, two argued against. Three RCTs listed arguments for fusion with instrumentation and two RCTs against this surgical procedure.

### Decompression

Arguments for decompression surgery emphasize the high success rates of decompression (n = 3) [11,13,16]. The main argument against a decompression alone was an increase or cause of vertebral instability, respectively the spondylolisthesis progression after decompression alone (n = 3) [12,15,16]. One RCT further argued that the continuous motion of the stenotic segments might compress the nerve roots as well as “produce osteophytes” (n = 1) [12].

### Decompression and fusion

Most authors argued for posterolateral fusion because this technique is “done frequently” and well established (n = 2) [10,14], shows excellent results for degenerative spondylolisthesis (n = 1) [13], has a high observed fusion rates (n = 1) [10], and shows a significant improvement in clinical outcome (n = 1) [9]. The arguments against a posterolateral fusion were that less invasive procedure used for decompression leave the relative stability of the spine undisturbed (n = 1) [12], that degenerative changes (osteophytes, decreased disc height, calcified ligaments) increase the stability of the spine and thereby decreasing the need for an arthrodesis (n = 1) [12], and that the indications for fusion in spinal stenosis and degenerative lumbar spondylolisthesis have remained unclear (n = 1) [11].

### Decompression and fusion with instrumentation

Arguments in favor of fusion with instrumentation included: increased fusion rate (n = 2) [10,14], prevention of spondylolisthesis progression (n = 1) [10], and a high fusion rate and a high level of patient satisfaction in 360° (circumferential) fusion (n = 1) [14]. Some authors quoted that fusion with instrumentation may improve fusion rate (n = 1) [9], may reduce rehabilitation time (n = 1) [9] and may improve patient outcome (n = 1) [9]. Further, that 270° fusion may be effective (n = 1) [14], that pedicle screw fixation increases rigidity despite resection of the posterior elements (n = 1) [10], and that semirigid systems have been advocated of obtaining spinal stability without sacrificing vertebral body bone density (n = 1) [10]. Authors that argued against instrumented fusion highlighted potential associated complications (n = 1) [10] as device related osteoporosis. Additionally, they quote the costs and use of great health care resources of the 360° (circumferential) procedure (n = 1) [14].

## Discussion

### Main findings

In this review of arguments for or against choosing a specific type of treatment for a patient with lumbar spinal stenosis nine randomized trials (RCTs) were appraised. The main argument for decompression surgery only was a high success rate (three RCTs). Furthermore, decompression alone is less invasive than fusion surgery (one RCT) and maintains the relative stability of the spine (one RCT). The main argument for fusion was that it is a well-established technique with a high fusion success rate (two RCTs) and statistically significant improvement in clinical outcome compared to decompressive lumbar laminectomy alone (one RCT). Arguments for the choice of fusion with instrumentation were an increased fusion rate compared to fusion (two RCTs) and prevention of spondylolisthesis progression (two RCTs).

Main arguments against decompression alone were an increase in vertebral instability and a progression of spondylolisthesis (three RCTs). The main argument against fusion was that the indication for fusion in spinal stenosis has remained unclear (one RCT). In particular, degenerative changes (osteophytes, decreased disc height, calcified ligaments) increase the stability of the spine and thereby reduce the need for arthrodesis (one RCT). The main arguments against fusion with instrumentation were that the invasive procedures are associated with more complications as, e.g., device related osteoporosis (one RCT) and are more expensive (one RCT).

### Comparison with the literature

The main argument identified in this appraisal for and against decompression alone in patient with lumbar spinal stenosis was whether or not instability should be treated with (instrumented) fusion procedures. Increased vertebral instability may lead to progression and compression of nerve roots and therefore require reoperation. Further, by reading the original papers we noticed that key terms including instability as well as reoperation are not clearly and unambiguously defined. Authors of different papers used various definitions for these terms. For the interpretation of study results and the appraisal of the clinical implications, or the synthesis of the results of original studies in a systematic review it is necessary to know what the different terms denote and how the different concepts, e.g., instability, are operationalized and quantified.

Various definitions of the term instability have been published that vary among experts. The meaning is different for clinicians, radiologists, and bioengineers [18]. An example for a clinical definition of instability is presented by White and Panjabi [19,20] as follows: “The loss of the spine’s ability to maintain its pattern of displacement under physiologic loads so there is no initial or additional neurologic deficit, no major deformity, and no incapacitating pain”. A radiological definition reported by Sonntag and Marciano [21]: “Increased angulatory or translatory motion noted on active flexion-extension lateral or anteroposterior bending radiographs that exceeds 4 mm or 10° angulation […]” According to Frymoyer [22] from a bioengineers point of view segmental instability is defined as “a loss of spinal motion segment stiffness, such that force application to that motion segment produces greater displacement than would be seen in a normal structure, resulting in a painful condition, the potential for progressive deformity, and neurologic structures at risk”.

And even between physicians of the same discipline, opinions on what instability means, differ exemplified by a further definition from radiology reported by White and Panjabi [19]. They defined criteria for diagnosing instability from flexion-extension radiographs as “sagittal plane translation greater than 4.5 mm or greater than 15% of the vertebral body width, or sagittal plane rotation of greater than 15° at L1/L2, L2/L3 or L3/L4, greater than 20° at L4/L5, or greater than 25° at L5/S1” [23].

An additional hurdle is that the association between clinical signs and radiological findings remains controversial and challenging [18,24]. A study conducted by Pitkanen et al. [25] found only poor correlation between radiological abnormalities identified in functional radiographs and clinical signs suggesting lumbar instability. It remains unclear whether the radiological approach is poor, or the definition of clinical instability is not that appropriate [25].

Recently an international and interdisciplinary expert panel was unable to agree on a definition of instability and its clinical relevance in symptomatic lumbar degenerative spondylolisthesis [26]. As a consequence of a lack of a broadly accepted definition and method of quantification of instability, researchers use different methods to quantify instability. This impedes the appraisal of the clinical impact of study results and hinders the synthesis of results from original studies in a systematic review. There is a need for a broadly accepted definition to facilitate the meaningful interpretation of study results.

An important question that needs to be addressed in future studies is whether a second surgery is necessary as a consequence of the first intervention or because of the progression of a preexisting degenerative disease. Many authors don’t define how they classify reoperations and the descriptions of reasons for repeat surgery vary [27], e.g., Martin et al. [28] defined in their study reoperation as “any lumbar operation in a patient who had at least 1 previous lumbar spine procedure. It was not necessarily a repeat of the same procedure or performed at the same vertebral location, but in all cases, it was still within the lumbar region”.

For the interpretation of study results it is important for clinicians and researchers that the reasons for a follow-up surgery in studies are clearly defined. Early reoperation can be necessary because of a surgical complication, such as spinal fluid leak, hematoma, infection, neurologic deficit, or mislocated instrumentation. For a reader these reoperations are clearly related to the prior surgery. This relation is less evident at a later stage. Non-union or complications resulting from surgical implants can be a reason for a reoperation at a later stage. Nevertheless, reoperations at a later stage may be performed because of recurrent or persistent pain symptoms, pseudoarthrosis, or progressive degeneration at another spine level [28]. Therefore, a detailed description, classification or definition is important to compare the results of different studies.

### Limitations

The main limitation of this study is the small number of randomized controlled trials comparing surgical procedures and therefore limited discussion on arguments for or against specific techniques. While spinal stenosis is a prevalent disease in elderly patients and surgical interventions are performed on a regular base only nine RCTs were available for the current appraisal.

Another limitation of this study is the missing distinction of different types of decompression procedures used (e.g. complete facetectomy or complete laminectomy). The extent of laminectomy or facetectomy may influence postoperative instability and should be addressed in all studies.

### Implication for research

It is important that researchers and clinicians agree on definitions for important key concepts such as instability and description of reasons for reoperation. Clear definitions of concepts and methods for the quantification or categorization facilitate the interpretation of study results and the synthesis of the results of different studies.

### Implication for clinical practice

For clinicians it is important to know when to recommend a certain intervention to their patient. The lack of high quality RCTs that addresses these important clinical questions impedes clinicians from an evidence-based treatment algorithm in patients with lumbar spinal stenosis. Indications for conservative or surgical intervention are oftentimes based on clinical judgment of the treating physician and depend on personal beliefs and experiences. Future research is needed to provide more robust evidence for such treatment choices.

## Conclusion

The main argument identified in this appraisal for and against decompression alone in patient with lumbar spinal stenosis was whether or not instability should be treated with (instrumented) fusion procedures. However, there is disagreement on how instability should be defined. In a first step it is important that researchers and clinicians agree on definitions for important key concepts such as instability and reoperations.

## Additional file

Additional file 1:
**Overview surgical procedure, follow-up, and author’s conclusion.**

## Abbreviations

RCTs: Randomized controlled trials
SR: Systematic review
LSS: Lumbar spinal stenosis
ALIF: Anterior lumbar interbody fusion
PLIF: Posterior lumbar interbody fusion
TLIF: Transforaminal lumbar interbody fusion
XLIF: Extreme lateral interbody fusion
