# Supplementary material for: Arguments for the choice of surgical treatments in patients with lumbar spinal stenosis – a systematic appraisal of randomized controlled trials
Source: BMC Musculoskelet Disord. 2015 Apr 22;16:96. doi: 10.1186/s12891-015-0548-8 (PMC4409719; doi:10.1186/s12891-015-0548-8)
Supplement: Additional file 1: — Overview surgical procedure, follow-up, and author’s conclusion. [file 12891_2015_548_MOESM1_ESM.docx]

Additional file 1: Overview surgical procedure, follow-up, and author’s conclusion

| **Study** | **Year** | **Intervention group** | **Control group** | **Follow**  **up (d)** | **Inclusion criteria** | **Author's conclusion** | **Definition of instability** |
| --- | --- | --- | --- | --- | --- | --- | --- |
| Bridwell [[17](#_ENREF_17)] | 1993 | Group 2: posterolateral fusion (autogenous bone graft) (n=10)  Group 3: instrumented posterolateral fusion (pedicle fixation) fusion (autogenous bone graft) (n=24) | Group 1: decompression alone (n=9) | 720 –  2580 | Spinal claudication caused by spinal stenosis at the spondylolisthesis level | A significant percentage of patients with degenerative spondylolisthesis undergoing decompression for spinal claudication have the potential to progress their listhesis. In our patient population, decompression with in situ fusion did not prevent spondylolisthesis progression, nor did it reliably fuse the segment. Properly performed pedicle fixation at the level of the spondylolisthesis did prevent spondylolisthesis progression, maintain preoperative sagittal alignment, and result in a high fusion rate. | Not reported |
| Fischgrund [[9](#_ENREF_9)] | 1997 | decompressive laminectomy and single level bilateral lateral autogenous intertransverse process arthrodesis with transpedicular instrumentation (instrumented posterolateral fusion) (n=35) | decompressive laminectomy and single level bilateral lateral autogenous intertransverse process arthrodesis (posterolateral fusion) (n=33) | 720 | Degenerative spondylolisthesis and spinal stenosis | In patients undergoing single-level posterolateral fusion for degenerative spondylolisthesis with spinal stenosis, the use of pedicle screws may lead to a higher fusion rate, but clinical outcome shows no improvement in pain in the back and lower limbs. | Not reported |
| Grob [[12](#_ENREF_12)] | 1995 | Group 2: decompression (with laminotomy and medial facetectomy) with arthrodesis (instrumented) mono-segmental (n=15)  Group 3: decompression (with laminotomy and medial facetectomy ) with arthrodesis (instrumented) multi-segmental (n=15) | Group 1: decompression alone (with laminotomy and medial facetectomy) (n=15) | 720 –  1080 | Degenerative lumbar spinal stenosis | We therefore believe that, in the absence of segmental instability, arthrodesis is not necessary after decompression of the lumbar spine in patients who have degenerative lumbar spinal stenosis, provided that the stabilizing posterior elements of the spine are preserved during the operation. | Instability was diagnosed on the basis of (1) a concomitant slip of a vertebra of more than five millimeters or another gross deformity such as rotational instability characterized by more than five millimeters of lateral offset on the anteroposterior roentgenogram or degenerative scoliosis, (2) spondylolysis with an osseous defect of the pars interarticularis, or (3) a previous operation on the lumbar spine |
| Herkowitz [[11](#_ENREF_11)] | 1991 | decompressive laminectomy and bilateral lateral intertransverse-process arthrodesis (posterolateral fusion) (n=25) | decompressive laminectomy (n=25) | 840 –  1440 | Degenerative spondylolisthesis and spinal stenosis | The results of this prospective study clearly demonstrate that decompressive lumbar laminectomy with intertransverse process arthrodesis is the operative procedure of choice for patients who have spinal stenosis associated with degenerative lumbar spondylolisthesis at single level. | Not reported |
| Kitchel [[13](#_ENREF_13)] | 2002 | instrumented posterolateral fusion (pedicle system) (autogenous bone graft) and posterior interbody fusion (machined allograft) (n=30) | instrumented posterolateral fusion (pedicle system) (autogenous bone graft) (n=32) | >720 | Grade 1/2 degenerative spondylolisthesis and accompanying spinal canal stenosis | The authors do not believe PLIF with allograft is indicated in patients over 60 years of age with Grade 1 or Grade 2 degenerative spondylolisthesis. | Not reported |
| Postacchini [[15](#_ENREF_15)] | 1993 | Group 1: multiple laminotomy (n=26) | Group 2: scheduled multiple laminotomy, converted to total laminectomy (n=9)  Group 3: total laminectomy (n=32) | 792 –  1908 | Central lumbar stenosis | Multiple laminotomy is recommended for all patients with developmental stenosis and for those with mild to moderate degenerative stenosis or degenerative spondylolisthesis. Total laminectomy is to be preferred for patients with severe degenerative stenosis or marked degenerative spondylolisthesis. | Not reported |
| Schoffer-  man [[14](#_ENREF_14)] | 2001 | 360° or circumferential fusion (ALIF plus transpedicular instrumentation plus posterolateral fusion (PLF) (autogenous bone graft)) (TSRH system plus allograft ring) (n=26, spinal stenosis n=5) | 270° fusion (ALIF plus transpedicular instrumentation without PLF) (TSRH system plus allograft ring) (n=22, spinal stenosis with facetectomy n=4) | 720 –  1350 | Structural problem: severe spinal stenosis, degenerated disc, discogenic pain, spondylolisthesis, scoliosis | Both the 360° and 270° fusions significantly reduce pain and improve function, and there are no significant clinical differences between them. However, there were shorter operating times, less blood loss, lower costs, and less utilization of health care resources associated with the 270° fusions. | Not reported |
| Thomé [[16](#_ENREF_16)] | 2005 | Group 1: bilateral laminotomy (n=40)  Group 2: unilateral laminotomy for bilateral decompression (n=40) | Group 3: laminectomy (n=40) | 360 | symptoms of neurogenic claudication or radiculopathy; radiological/neuroimaging evidence of degenerative lumbar stenosis; absence of associated pathological entities such as disc herniations or instability; no history of surgery for lumbar stenosis or lumbar fusion | Bilateral and unilateral laminotomy allowed adequate and safe decompression of lumbar stenosis, resulted in a highly significant reduction of symptoms and disability, and improved health-related quality of life. Outcome after unilateral laminotomy was comparable with that after laminectomy. In most outcome parameters, bilateral laminotomy was associated with a significant benefit and thus constitutes a promising treatment alternative. | Spinal instability was defined as sagittal-plane translation of 5 mm or more documented on flexion–extension radiography. |
| Zdeblick [[10](#_ENREF_10)] | 1993 | instrumented posterolateral fusion (autogenous bone graft)  Group 1: TSRH system (rigid pedicle screw/rod fixation) (n=37, 8 with stenosis)  Group 2: Luque II system (semirigid pedicle screw/plate fixation) (n=35, 3 with stenosis) | posterolateral fusion (autogenous bone graft) (n=52, 8 with stenosis) | 270 –  840 | Degenerative conditions of the spine: isthmic spondylolisthesis, degenerative disc disease, degenerative spondylolisthesis, degenerative scoliosis with spinal stenosis, repair of previous lumbar pseudarthrosis) | Rigid pedicle screw/rod fixation led to a significantly higher percentage of fusions in degenerative lumbar disease than did fusion without instrumentation. The fusion rate was also higher with rigid instrumentation than with semirigid plate/screw fixation, although clinical results were improved with both fixation systems. I recommend the use of a rigid pedicle screw instrumentation system in patients undergoing fusion for degenerative disc disease or degenerative spondylolisthesis, and in revision surgery. | Not reported |
